# Influenza B virus non-structural protein 1 counteracts ISG15 antiviral activity by sequestering ISGylated viral proteins

**DOI:** 10.1038/ncomms12754

**Published:** 2016-09-02

**Authors:** Chen Zhao, Haripriya Sridharan, Ran Chen, Darren P. Baker, Shanshan Wang, Robert M. Krug

**Affiliations:** 1 Department of Molecular Biosciences, Center for Infectious Disease, Institute for Cellular and Molecular Biology, University of Texas at Austin, Austin, Texas 78712, USA; 2 Biogen, Inc., Cambridge, Massachusetts 02142, USA

## Abstract

The ubiquitin-like protein ISG15 and its conjugation to proteins (ISGylation) are strongly induced by type I interferon. Influenza B virus encodes non-structural protein 1 (NS1B) that binds human ISG15 and provides an appropriate model for determining how ISGylation affects virus replication in human cells. Here using a recombinant virus encoding a NS1B protein defective in ISG15 binding, we show that NS1B counteracts ISGylation-mediated antiviral activity by binding and sequestering ISGylated viral proteins, primarily ISGylated viral nucleoprotein (NP), in infected cells. ISGylated NP that is not sequestered by mutant NS1B acts as a dominant-negative inhibitor of oligomerization of the more abundant unconjugated NP. Consequently formation of viral ribonucleoproteins that catalyse viral RNA synthesis is inhibited, causing decreased viral protein synthesis and virus replication. We verify that ISGylated NP is largely responsible for inhibition of viral RNA synthesis by generating recombinant viruses that lack known ISGylation sites in NP.

## Abstract (web summary)

The ubiquitin-like protein ISG15 can be covalently linked to cellular and viral proteins, but the consequences of this ‘ISGylation' remain largely unknown. Here, Zhao *et al.* show that ISGylation of the influenza B virus nucleoprotein inhibits formation of a functional viral replication complex.

## Graphical abstract

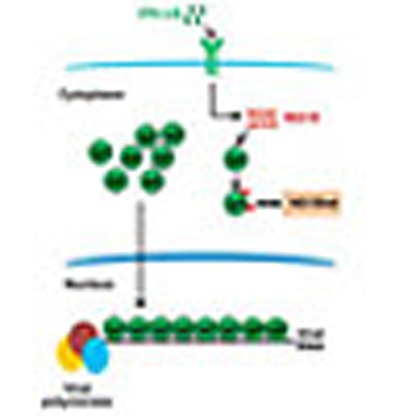

ISG15 is an ubiquitin-like protein (Ubl) that is highly induced by the interferon (IFN)-α/β produced during infection of human cells by viral and bacterial pathogens^1^. ISG15 comprises two Ubl domains connected by a short five-amino acid linker. Unlike ubiquitin, the sequence of ISG15 varies between species^2^. ISG15 is covalently conjugated via its C-terminal LRLRGG sequence to target proteins by the sequential action of three enzymes that are also induced by IFN-α/β, namely, E1 (UbE1L), E2 (UbcH8) and E3 (Herc5; ref. 3). ISG15 conjugation (ISGylation) can be reversed by the IFN-α/β-induced isopeptidase (USP18) that cleaves ISG15 from target proteins^4^. The finding that several viruses encode proteins that either bind ISG15 or cleave ISG15 from target proteins indicates that ISGylation and/or free ISG15 have antiviral functions^5^. The non-structural protein 1 of influenza B virus (NS1B) was the first viral protein that was shown to bind ISG15 (ref. 6). The E3 protein of vaccinia virus also interacts with ISG15 (ref. 7). Several viruses, nairoviruses, arteriviruses and severe acute respiratory syndrome virus, encode enzymes that can remove ISG15 from its targeted proteins^8^.

Studies using knockout mice demonstrated that ISGylation protects mice against lethal infection by several viruses, including influenza A and B viruses^9^,^10^,^11^. ISGylation also inhibits influenza A virus replication in human tissue culture cells^12^,^13^,^14^. This inhibition results from the ISGylation of one or two lysines (Ks) of the viral NS1A protein, leading to the loss of NS1A function^13^,^14^. For example, ISGylation of the K at position 41 in the NS1A protein of a 1972 H3N2 virus led to a loss of interaction with the cellular protein, importin-α, that mediates nuclear import of the NS1A protein^13^. A recombinant virus that encodes a NS1A protein in which K at position 41 was replaced with an arginine (R) showed tenfold increased resistance to IFN-β pretreatment compared with wild-type (wt) virus, indicating that ISGylation of NS1A at residue K41 contributes to the IFN-β sensitivity of influenza A virus. The ISGylation of NS1A viral protein is consistent with the finding that ISGylation targets newly synthesized proteins because viral proteins constitute the majority of the newly synthesized proteins in virus-infected cells^15^. However, it is not known how ISGylated NS1A, which constitutes <5% of the total NS1A protein in infected cells, leads to the loss of function of the whole NS1A protein population. One hypothesis is that such a low level of an ISG15-conjugated viral protein would have a dominant-negative effect on the function of its unconjugated counterpart if the viral protein functions as an oligomer in infected cells^5^,^15^. However, it has not been established that ISGylated NS1A proteins, nor ISGylated proteins of any virus operate in a dominant-negative manner to inhibit oligomerization of their unconjugated counterparts in virus-infected cells.

Unconjugated (free) ISG15 has also been shown to have a role in host defense against pathogens. Free extracellular ISG15 protects mice against Chikungunya virus infection, but not by inhibiting virus replication, but rather by downregulating the expression of virus-induced proinflammatory cytokines^16^. Other investigators have shown that free extracellular ISG15 can have the opposite effect, namely, stimulating IFN-γ production in human lymphocytes, leading to increased cytokine production. Patients with an inherited ISG15 deficiency were more susceptible to mycobacterial disease caused by little or no activation of IFN-γ, indicating that ISG15 is required for antibacterial immunity^17^. In contrast, these investigators found that cells from these patients were not more susceptible to infection by several viruses. A subsequent study of ISG15-deficient patients and cells derived from these patients showed that intracellular free ISG15 in human cells downregulates the IFN-α/β response by binding and stabilizing USP18, a protein with two activities, removal of ISG15 from ISGylated proteins and inhibition of IFN signalling^18^,^19^. A recent study analysed the effect of a relatively short (12 h) treatment with IFN-α/β followed by a 36 h ‘rest period' on the antiviral state of ISG15-negative and ISG15-positive human cells^20^. The levels of replication of several viruses were significantly higher in the ISG15-positive cells. This increase in replication was attributed to the downregulation of IFN-α/β signalling resulting from the stabilization of USP18 by free ISG15. These investigators also found that murine ISG15 does not bind USP18, and hence does not downregulate IFN-α/β signalling in mice. Based on these results, these investigators proposed that free intracellular ISG15, as well as free secreted ISG15 mediate the major biological activities of ISG15 pathway in humans, and that ISGylation does not have a documented role in human antiviral activity^18^,^20^. This proposal is contrary to previous studies, which established that ISGylation of the influenza A virus NS1A protein causes the inhibition of replication of influenza A virus in human cells^13^,^14^.

Influenza B virus provides an appropriate model for determining how human ISG15 and its conjugation onto target proteins affect virus replication in human cells. Unlike influenza A viruses, influenza B viruses infect only humans^21^. This limited host range might be attributed to the NS1B protein of influenza B virus, which binds only human and non-human primate ISG15 molecules, demonstrating that the NS1B protein can only block the antiviral functions of ISG15 in human and non-human primate cells^2^,^22^. The structure of human ISG15 in complex with a N-terminal fragment of the NS1B protein showed the basis for this species specificity: the NS1B protein-binding site on ISG15 includes the two N-terminal residues of the linker region that are specific for human and non-human primate ISG15s (ref. 23). This structure also identified two NS1B amino acids that are required for binding ISG15, tryptophan (W) at position 36 and glutamine (Q) at position 37. A mutant NS1B protein with alanine (A) substitutions at positions 36 and 37 did not bind ISG15. In addition, unlike the wt NS1B protein, the mutant NS1B protein did not inhibit IFN-β-induced ISGylation as assayed in transfection experiments^23^.

In the present study, we generate a recombinant influenza B virus that encodes a NS1B protein defective in ISG15 binding due to alanine substitutions at positions 36 and 37. Using this mutant virus, we show that contrary to the transfection result described above, the NS1B protein does not inhibit ISGylation in influenza B virus-infected cells. Instead, the NS1B protein binds and sequesters ISGylated viral proteins, especially ISGylated viral nucleoproteins (NPs). We demonstrate that ISGylated NP proteins that are not sequestered by the mutant NS1B protein act in a dominant-negative manner in mutant virus-infected cells to inhibit oligomerization of the more abundant unconjugated NP proteins. As a result, formation of viral ribonucleoproteins (RNPs) that catalyse viral RNA synthesis is inhibited, causing decreased production of viral RNAs and viral proteins, and hence decreased virus replication. Our results establish that IFN-α/β-induced ISGylation can exhibit potent antiviral activity in human cells.

## Results

### NS1B does not inhibit ISGylation in virus-infected cells

**Figure 1:**
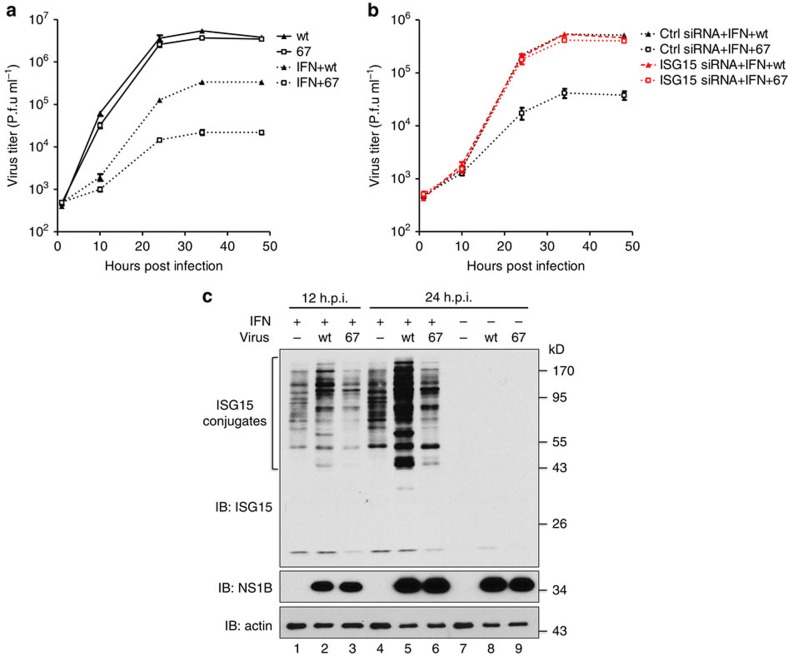
The NS1B protein does not inhibit IFN-β-induced ISGylation in influenza B virus-infected cells. (**a**) Multiple cycle growth curves of wt and 67 mutant virus in A549 cells with or without IFN-β pretreatment. Where indicated, A549 cells were treated with 1,000 IU ml^−1^ of human IFN-β for 18 h before virus infection. Cells were infected with 0.1 p.f.u. per cell of wt virus or 67 mutant virus. Error bars show the s.d. of triplicate assays of virus titers at the indicated times of infection determined by plaque assays in MDCK cells. (**b**) The effect of siRNA knockdown of ISG15 on the multiple cycle growth of wt and 67 mutant virus in IFN-β pretreated A549 cells. Where indicated, ISG15-specific siRNA was transfected into cells for 24 h before IFN-β treatment. The efficiency of the ISG15-specific siRNA in inhibiting ISGylation is shown in Fig. 4a, lanes 5 and 6. Error bars show the s.d. of triplicate assays of virus titers at the indicated times of infection. See also (Supplementary Fig. 1a,b). (**c**) The effect of wt and 67 mutant virus on IFN-β-induced ISGylation. A549 cells were treated with 1,000 IU ml^−1^ of human IFN-β for 12 h or were not treated with IFN. Cells were then either mock infected, or infected with 5 p.f.u. per cell of wt or 67 mutant virus. Cell extracts isolated at indicated times after infection or mock infection were analysed by immunoblots probed with the indicated Abs.

To determine whether NS1B inhibits ISGylation in virus-infected cells, we generated a recombinant influenza B virus that expresses a NS1B protein with W36A and Q37A mutations. This recombinant virus, denoted as the 67 mutant virus, replicated at the same rate and to the same extent as wt virus during multiple cycle growth in human A549 cells (Fig. 1a). In contrast, the 67 mutant virus was inhibited to a greater extent than wt virus when A549 cells were pretreated with IFN-β. Whereas IFN-β pretreatment of A549 cells reduced wt virus replication 20–30-fold, replication of the 67 mutant virus was reduced 200–300-fold. This tenfold difference was eliminated by an ISG15-specific short interfering RNA (siRNA), but not the control siRNA (Fig. 1b). A different ISG15-specific siRNA had the same effect (Supplementary Fig.1a,b). These results establish that the primary defect of the 67 mutant virus is the inability to counteract the antiviral activity of IFN-β-induced ISG15.

To determine the effect of the 67 mutant virus on IFN-β-induced ISGylation, A549 cells were treated with IFN-β for 12 h followed by infection with 5 plaque-forming units (p.f.u.)  ml^−1^ of wt or 67 mutant virus. This duration and dosage of IFN-β pretreatment was sufficient to induce free ISG15 but no detectable ISGylation at the time of virus infection. Consequently, ISGylation occurs only during virus infection, enabling us to determine the effects of wt and 67 mutant virus on ongoing ISGylation. Infected cells were harvested at 12 and 24 h after infection, and the amount of ISG15 conjugates was measured using immunoblots. Based on the previously published co-transfection experiment^23^, it would be expected that wt virus, but not the 67 mutant virus, would inhibit ISGylation. Surprisingly, we observed the opposite phenotype: substantially more ISG15 conjugates were produced in wt virus-infected cells than in 67 mutant virus-infected cells (Fig. 1c). These results lead to the unexpected conclusion that the NS1B protein does not inhibit IFN-β-induced ISGylation in influenza B virus-infected cells.

ISGylation occurs only when IFN-β treatment precedes influenza B virus infection. Wt virus infection in the absence of IFN-β pretreatment induces ISG15, but not ISGylation (lane 8), which is more clearly shown in Supplementary Fig. 2a. ISGylation is not induced because virus infection alone does not induce the synthesis of UbcH8, the E2 enzyme for ISGylation, and probably the other ISG15 conjugation enzymes. When IFN-β was added after infection, both the wt and 67 viruses inhibited the synthesis of UbcH8 and hence ISGylation (Supplementary Fig. 2b).

### Wild-type NS1B binds ISG15 conjugates during virus infection

**Figure 2:**
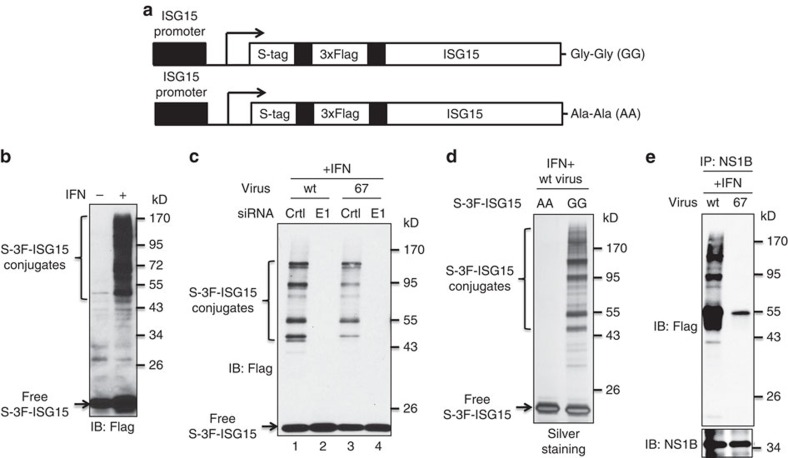
The wt NS1B protein, but not the 67 mutant NS1B protein, binds and sequesters ISG15 conjugates in IFN-β-pretreated influenza B virus-infected cells. (**a**) Schematic representation of the two IFN-β-inducible, S-3F-tagged ISG15 sequences inserted into A549 cells. (**b**) S-3F-ISG15 A549 cells were either untreated or treated with 1,000 IU ml^−1^ of human IFN-β for 24 h. Cell extracts were analysed by immunoblots probed with Flag Ab. (**c**) S-3F-ISG15 A549 cells were transfected with control (ctrl) siRNA or E1(UbelL)-specific siRNA for 24 h, followed by treatment with 1,000 IU ml^−1^ of human IFN-β for 12 h. Cells were then infected with 10 p.f.u. per cell of either wt or 67 mutant virus for 18 h. Cell extracts were analysed by immunoblots probed with Flag Ab. (**d**) S-3 F-ISG15 mut or wt cells were treated with 1,000 IU ml^−1^ of human IFN-β for 16 h, and then infected with 10 p.f.u. per cell of wt virus for 24 h. Denatured cell extracts were subject to double-affinity purification using anti-Flag M2 agarose and S-protein agarose. The purified proteins were detected by silver staining after SDS page. (**e**) The cell extracts that were analysed in lanes 1 and 3 of panel **c** were instead immunoprecipitated with NS1B Ab, and the immunoprecipitates were then analysed by immunoblots probed with Flag Ab and NS1B Ab.

We further analysed the interactions of the NS1B protein with ISG15 and ISG15 conjugates in virus-infected cells. To facilitate this analysis, we generated two A549 cell lines that express either wt ISG15 or an ISG15 mutant containing C-terminal Ala-Ala instead of the Gly-Gly required for ISG15 conjugation, each ISG15 N-terminally tagged with both S.tag and 3xFlag tag under the control of the ISG15 promoter. These cell lines are denoted as ISG15GG and ISG15AA cells (Fig. 2a). In ISG15GG cells, the double-tagged ISG15 protein is effectively conjugated to target proteins in an IFN-β-dependent manner (Fig. 2b). As is the case for endogenous ISG15, a larger amount of conjugates containing this double-tagged ISG15 was produced in wt virus-infected cells than in 67 virus-infected cells (Fig. 2c). SiRNA knockdown of the E1 enzyme (Ube1L) eliminated these conjugates, verifying that they are ISG15 conjugates. As further verification, we showed that these conjugates were not produced during wt virus infection of the IFN-treated ISG15AA cells (Fig. 2d). To determine whether the ISG15 conjugates in wt and 67 virus-infected cells are bound to the NS1B protein, the cell extracts from ISG15GG cells infected with wt virus (Fig. 2c, lane 1) and with 67 virus (Fig. 2c, lane 3) were immunoprecipitated with NS1B antibody, followed by immunoblots probed with Flag and NS1B antibodies (Fig. 2e). The result showed that wt NS1B protein, but not the 67 mutant NS1B protein, binds ISG15 conjugates.

### NP protein is ISGylated in influenza B virus-infected cells

**Figure 3:**
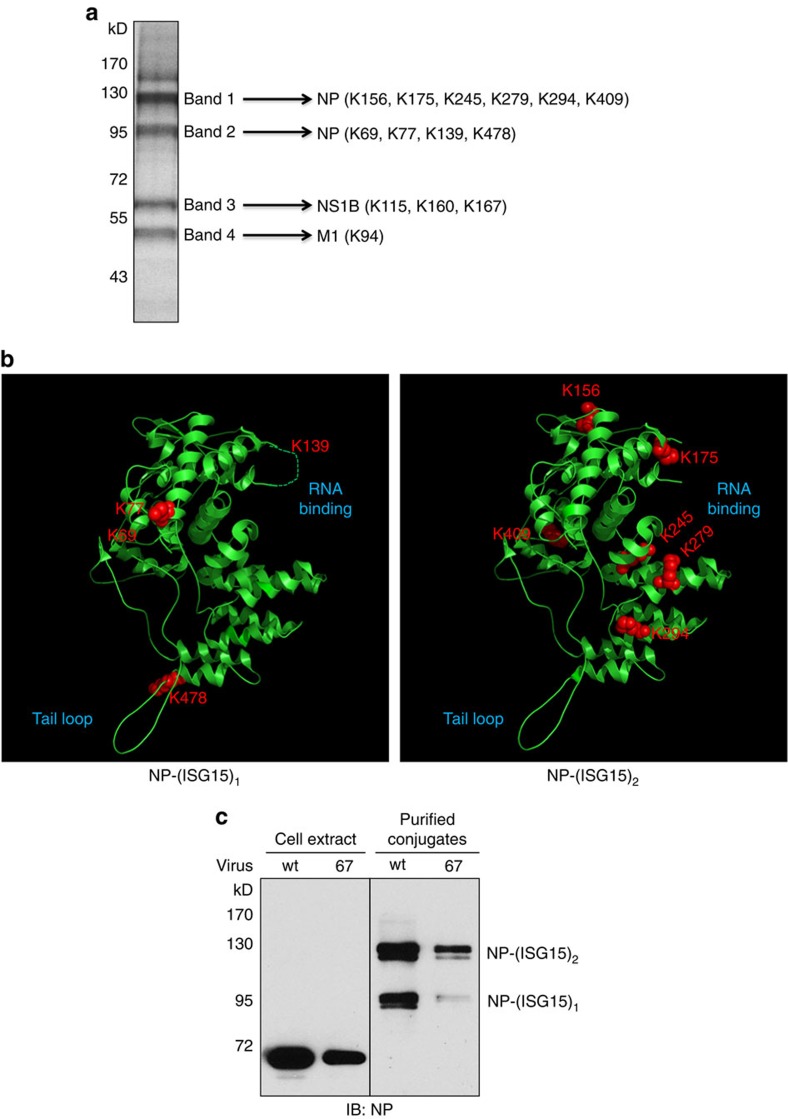
The major target of ISGylation in influenza B virus-infected cells is NP. (**a**) Colloidal blue-stained gel of ISG15 conjugates purified by double-affinity selection from S-3F-ISG15GG A549 cells which were pretreated with IFN-β and then infected with wt virus for 24 h. The four bands that were analysed by mass spectrometry and the ISG15 conjugation sites that were identified in these four bands are shown. See also Supplementary Table 1. (**b**) Location of the ISG15-modified lysines (Ks) mapped onto the structure of influenza B virus NP (PDB accession code 3TJ0). (**c**) S-3F-ISG15GG A549 cells were pretreated with IFN-β and then infected with 10 p.f.u. per cell of wt or 67 mutant virus for 24 h. Cell extracts and ISG15 conjugates purified from these extracts by double-affinity selection were analysed by immunoblots probed with NP Ab.

To identify the ISGylation targets, we purified ISG15 conjugates from IFN-β-pretreated, wt virus-infected ISG15GG cells by sequential affinity purification on anti-Flag M2 affinity resin and S-protein agarose under denaturing conditions. The resulting purified proteins were separated by SDS–polyacrylamide gel electrophoresis into four major bands identified by Colloidal blue staining (Fig. 3a). Mass spectrometry of these bands showed that they contained primarily viral proteins, specifically NP in bands 1 and 2, NS1B in band 3, and matrix protein (M1) in band 4. Based on molecular weights, the NP species in bands 1 and 2 are most likely NP-(ISG15)_2_ and NP-(ISG15)_1_, respectively. Mass spectrometry also identified the lysines (Ks) that are modified by ISG15 in these three proteins (Fig. 3b and Supplementary Table 1). Multiple Ks of NP are ISG15 attachment sites, six Ks in NP-(ISG15)_2_ and four other Ks in NP-(ISG15)_1_. Figure 3b shows the location of these sites on the crystal structure of influenza B virus NP^24^. Some of these sites are located at or near important functional regions of NP: K478 is located near the tail loop that mediates oligomerization; K139, K175, K245 and K279 are located at or close to the electropositive groove required for RNA binding; and K69 and K77 are located in the N terminus that functions as a nuclear localization signal^25^.

NP-(ISG15)_1_ and NP-(ISG15)_2_ were also found in IFN-β-pretreated, 67 virus-infected cells at lower levels compared with wt virus-infected cells (Fig. 3c). The immunoblot of the input cell extracts did not detect these ISGylated NPs, whereas large amounts of unconjugated NP were detected, showing that ISGylated NPs comprise only a minor fraction of the total NP population in virus-infected cells.

### NS1B-ISG15 binding linked to viral RNA synthesis

**Figure 4:**
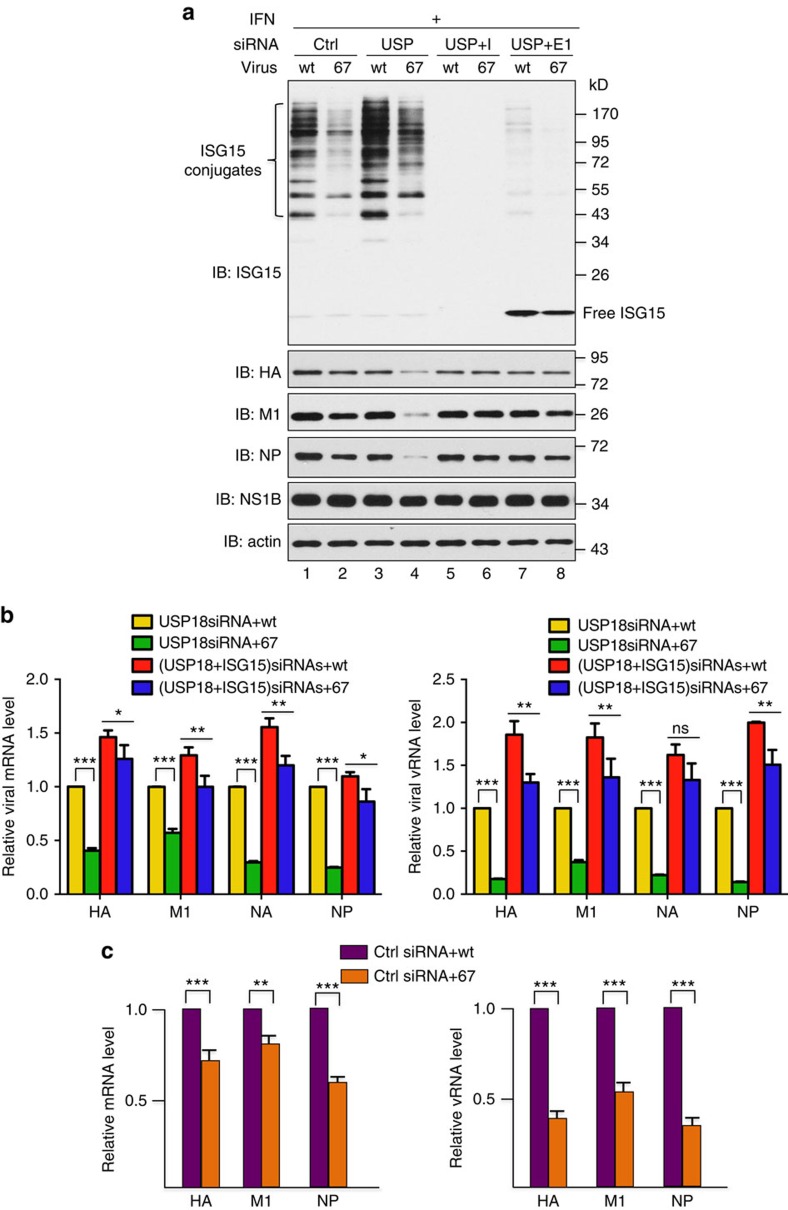
ISGylation is responsible for the inhibition of viral RNA production in 67 mutant virus-infected cells. (**a**) Viral protein synthesis in wt and 67 mutant virus-infected A549 cells expressing different levels of IFN-β-induced ISGylation. Cells were transfected with the indicated siRNAs (Ctrl: control siRNA; USP: USP18-specific siRNA; I: ISG15-specific siRNA; E1: Ube1L-specific siRNA) for 24 h, followed by 16 h of treatment with 1,000 IU ml^−1^ human IFN-β and subsequent infection with 5 p.f.u. per cell of wt or 67 mutant virus. At 24 h after infection, cells were collected, and extracts were analysed by immunoblots probed with the indicated Abs. Quantitation of immunoblots was performed using ImageJ software (NIH). See also (Supplementary Fig. 1c,d). (**b**) Viral RNA synthesis in wt and 67 mutant virus-infected A549 cells expressing different levels of IFN-β-induced ISGylation. Cells were transfected with the indicated siRNAs for 24 h, followed by 16 h of treatment with 1,000 IU ml^−1^of human IFN-β and subsequent infection with 5 p.f.u. per cell of wt or 67 mutant virus. At 24 h after infection, cells were collected, and the levels of the indicated viral mRNAs and vRNAs were determined by RT–PCR, normalized to the level of β-actin mRNA. The levels of viral mRNAs and viral vRNAs are shown as fold changes relative to the levels in the USP18 siRNA-treated, wt virus-infected sample. Results shown are mean +/− s.d. of three independent experiments, with each measurement performed in triplicate. *P* values were calculated using two-way analysis of variance (ANOVA) test. (**c**) Viral RNA synthesis in wt and 67 mutant virus-infected cells expressing endogenous levels of IFN-β-induced ISGylation. Cells were transfected with a control siRNA before IFN-β treatment followed by virus infection. Results shown are mean +/− s.d. of three independent experiments, with each measurement performed in triplicate. *P* values were calculated using two-way ANOVA test. ***P<0.001, **P<0.01, *P<0.05, ns *P*>0.05.

Because our results show that viral proteins are primary ISGylation targets in influenza B virus-infected cells, the defect(s) in the replication of the 67 mutant virus is likely attributable to the actions of the ISGylated NP, M1 and/or NS1B proteins that are not sequestered by the 67 mutant NS1B protein. As the first approach to identify which ISGylated viral protein is responsible for this defect(s), we determined the step(s) in virus replication that is inhibited by ISGylation in 67 virus-infected cells. IFN-β pretreated A549 cells were infected with either wt or 67 virus at high multiplicity to infect all cells, thereby generating a single cycle of virus replication. Before IFN-β treatment the cells were transfected with the siRNAs indicated in Fig. 4a. For the cells transfected with the control siRNA (lanes 1 and 2), the amounts of the viral haemagglutinin (HA), M1 and NP proteins produced in cells infected with the 67 virus were 30–40% lower than the amounts in cells infected with wt virus. Transfection of a USP18-specific siRNA (lanes 3 and 4) increased ISGylation and reduced the production of HA, M1 and NP proteins in 67 virus-infected cells by 60–85%, with little or no effect on the production of these viral proteins in wt virus-infected cells. A second USP18-specific siRNA had the same effect (Supplementary Fig. 1c,d). In contrast, the level of the NS1B protein showed no decrease in 67 virus-infected cells; a likely explanation for the lack of effect on NS1B protein synthesis is presented in Supplementary Fig. 3. The effect of USP18 knockdown on HA, M1 and NP production in 67 virus-infected cells was reversed by co-transfection of an ISG15-specific siRNA (lanes 5 and 6) or an E1-specific siRNA (lanes 7 and 8), either of which eliminated ISG15 conjugation, establishing that this effect is caused by augmented ISGylation. This result is consistent with a recent study that established the proviral activity of USP18 via its deconjugation of ISG15 (ref. 26). Because the free ISG15 that remained after E1 knockdown did not inhibit viral protein production in 67 virus-infected cells (lane 8), we conclude that ISGylated proteins, not free intracellular ISG15, are responsible for the inhibition of viral protein production in 67 virus-infected cells.

To determine whether ISGylation acts at the level of viral RNA synthesis, we measured the levels of several mRNAs (mRNAs for HA, M1, NA (neuraminidase) and NP) and the corresponding viral RNAs (vRNAs) in wt virus- and 67 virus-infected cells using quantitative reverse transcription PCR (RT–PCR). In cells in which USP18 was siRNA-silenced, the levels of all the mRNAs were 30–50% lower in 67 virus-infected cells than in wt virus-infected cells (Fig. 4b, left panel), mirroring the different levels of viral proteins under the same conditions (Fig. 4a, lanes 3 and 4). The reduction in vRNA levels was consistently larger (70–85%) (Fig. 4b, right panel). In cells in which ISG15 as well as USP18 were siRNA-silenced, the difference in mRNA and vRNA levels between wt and 67 virus-infected cells was reduced (Fig. 4b), similar to the effects on viral protein production (Fig. 4a, lanes 5 and 6).

In most cases, ISG15 knockdown did lead to a small but significant increase (less than twofold) in mRNA and vRNA production in wt virus-infected cells in which USP18 was also knocked down (Fig. 4b). This small increase may be due to the augmented levels of ISGylated viral proteins caused by USP18 knockdown. As a result of this augmentation, wt NS1B protein might not be capable of sequestering all the ISGylated viral proteins, leading to a small amount of antiviral activity caused by free ISGylated viral proteins.

We also determined whether mRNA and vRNA production was reduced in 67 virus-infected cells expressing endogenous levels of ISGylation. In cells transfected with a control siRNA (Fig. 4c), the reduction in mRNA levels mirrored the different levels of viral proteins produced by the 67 and wt viruses under the same conditions (compare Fig. 4c, left panel with Fig. 4a, lanes 1 and 2). Again, the reduction in vRNA levels was larger (45–65%) (Fig. 4c, right panel), and presumably accounts for the fourfold reduction in virus replication under these infection conditions (Supplementary Fig. 4). We conclude that ISGylation is responsible for the inhibition of viral RNA production in 67 virus-infected cells, leading to the inhibition of the production of viral proteins that are the primary targets for ISGylation, thereby explaining why the level of ISGylation is reduced in 67 virus-infected cells.

### ISGylated NP inhibits oligomerization of unconjugated NP

**Figure 5:**
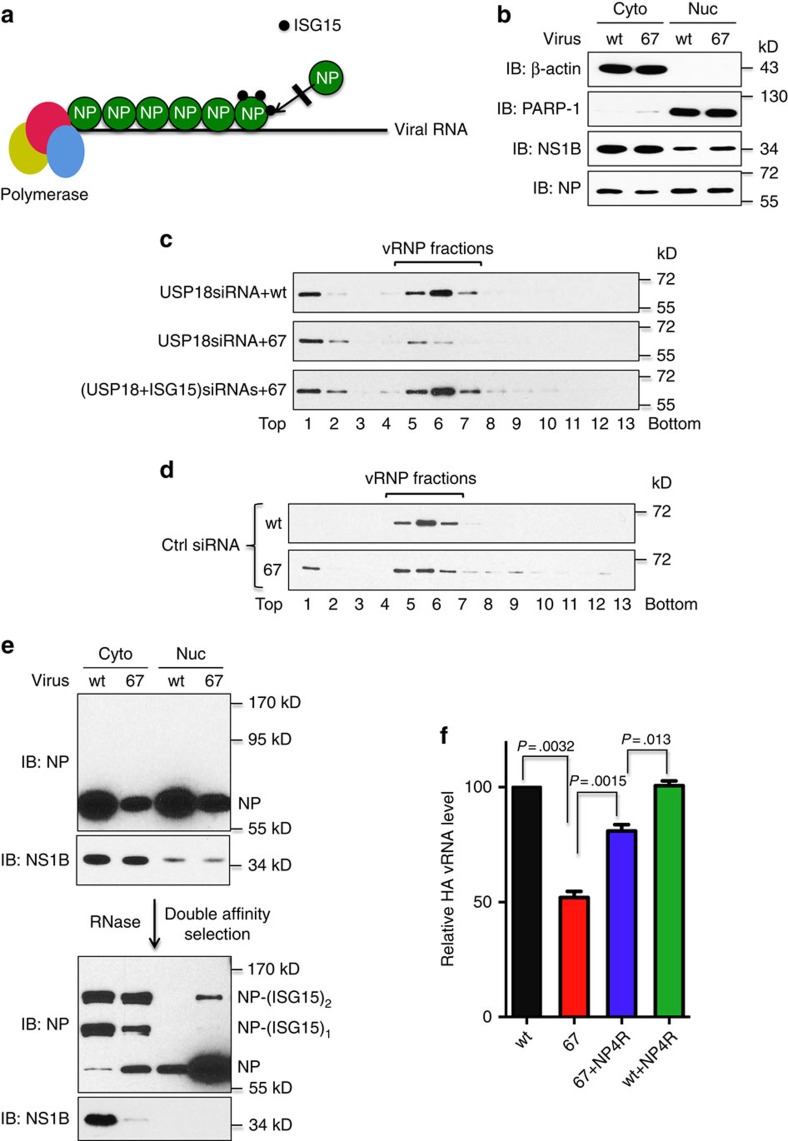
ISGylated NP inhibits NP oligomerization and hence formation of viral RNPs that are responsible for viral RNA synthesis. (**a**) Model of the termination of NP oligomerization by ISGylated NP. See also (Supplementary Fig. 6). (**b**) Fractionation of infected cells into cytoplasmic and nuclear fractions. (**c**) Glycerol gradient analysis of vRNP formation in the nucleus of infected cells. A549 cells were transfected with the indicated siRNAs, followed by 16-h treatment of IFN-β and subsequent infection with either wt or 67 mutant virus. At 16 h after infection, nuclear extracts were prepared and subjected to glycerol gradient ultracentrifugation. Fractions were collected and analysed by immunoblots probed with NP Ab. (**d**) Glycerol gradient analysis of vRNP formation in the nucleus of infected cells expressing endogenous ISGylation. A549 cells were transfected with a control siRNA, and were pretreated with IFN-β for 12 rather than 16 h before virus infection. (**e**) ISGylated NP in the nucleus of 67 mutant virus-infected cells is associated with a large number of unconjugated NP molecules. S-3F-ISG15GG A549 cells were pretreated with USP18 siRNA and IFN-β followed by infection with wt or 67 mutant virus. Cells collected at 16 h after infection were separated into cytoplasmic and nuclear fractions. ISG15 conjugates were purified from both fractions by double-affinity selection under non-denaturing conditions in presence of RNase, and were analysed by immunoblots probed with NP or NS1B Ab. (**f**) Elimination of a set of ISGylation site of NP largely relieved the IFN-β-mediated inhibition of vRNA synthesis in 67 virus-infected cells. A549 cells were treated with IFN-β for 16 h before infection with the indicated viruses. wt+NP4R and 67+NP4R represent, respectively, wt and 67 mutant virus expressing a NP mutant with K-to-R substitutions at position 156,245,279 and 478. At 24 h after infection, cells were collected, and the levels of the HA vRNA were determined by RT–PCR, normalized to the level of β-actin mRNA. Results shown are mean +/− s.d. of three independent experiments, with each measurement performed in triplicate. *P* values were obtained with two-tailed *t*-test. See also (Supplementary Fig. 5).

Of the three ISGylated viral proteins that are not sequestered by the 67 mutant NS1B protein, ISGylated NP is most likely responsible for the inhibition of viral RNA synthesis because NP is an integral component of the double-helical viral RNPs that catalyse viral RNA synthesis^27^,^28^. Viral RNPs are comprised of a single trimeric viral polymerase that binds to the 5′ and 3′ ends of each vRNA (negative-sense) segment and cRNA (positive-sense) segment, and multiple NP proteins that coat the entirety of the rest of the vRNA and cRNA chains^27^. The formation of vRNPs requires NP oligomerization along the vRNA and cRNA chains^28^,^29^. In this process, the tail loop of each NP molecule inserts into a cavity in the adjacent NP molecule^24^,^30^,^31^. Even the small amount of ISGylated NP molecules produced in virus-infected cells could disrupt NP oligomerization in a dominant-negative manner, if only the loop or the insertion site, but not both is blocked by ISG15-modified Ks (Fig. 5a). For example, an ISGylated NP might bind to an unconjugated NP at the end of a nascent NP oligomer because the tail loop of the unconjugated NP is able to insert into the pocket in the ISGylated NP that is not blocked by ISG15-modified Ks. In contrast, the tail loop of the ISGylated NP might be blocked by ISG15-modified Ks from inserting into the pocket of an unconjugated incoming NP, thereby terminating oligomerization. Indeed, for the NP protein of influenza A virus, a mutant with a defective tail loop and an intact insertion cavity, or vice versa, has been shown to inhibit wt NP-mediated vRNP formation in a dominant-negative manner^32^,^33^. We tested this hypothesis as described below.

Because the assembly of vRNPs during infection occurs in the nucleus, we determined whether nuclear vRNP formation is inhibited by ISGylated NP proteins in 67 virus-infected cells as compared with wt virus-infected cells. First, we devised a method for separating virus-infected cells into cytoplasmic and nuclear fractions. A549 cells pretreated with IFN-β were infected with either wt or 67 virus, and cell extracts collected at 16 h after infection were separated into cytoplasmic and nuclear fractions (Fig. 5b). As indicated by nuclear PARP-1 marker and cytoplasmic β-actin marker, there was no apparent cross contamination between the cytoplasmic and nuclear fractions. The majority of the NS1B protein was localized in the cytoplasmic fraction in cells infected with either virus, consistent with previously published immunofluorescence results that showed a shift of the NS1B protein from the nucleus to the cytoplasm during the course of infection^34^. NP was located in both the cytoplasm and nucleus.

To determine whether nuclear vRNP formation is inhibited in 67 virus-infected cells, A549 cells pretreated with USP18 siRNA and IFN-β were infected with wt or 67 virus, and nuclear extracts were analysed by glycerol gradient centrifugation to separate vRNPs from free NP molecules (Fig. 5c). Immunoblots probed with NP antibody showed that ∼75% of nuclear NP in wt virus-infected cells sedimented at the position of vRNPs (fractions 5-7). In contrast, only 30% of the NP in 67 virus-infected cells sedimented at the position of vRNPs, predominantly at the position of the smaller size vRNPs (fraction 5), consistent with a defect in vRNP assembly. When 67 virus-infected cells were treated with ISG15-specific siRNA, nuclear vRNP formation was restored to a level comparable to that in wt virus-infected cells. To verify that nuclear vRNP assembly is defective in 67 virus-infected cells, we devised treatment conditions (control siRNA and shorter IFN treatments) that resulted in the production of approximately equal amounts of nuclear NP in wt virus and 67 virus-infected cells. As shown in Fig. 5d, under these conditions all the nuclear NP in wt virus-infected cells was in vRNPs, whereas in 67 virus-infected cells 25% of the total NP was free at the expense of NP in vRNPs, confirming that vRNP formation in 67 virus-infected cells was inhibited relative to wt virus-infected cells.

The model for the termination of NP oligomerization shown in Fig. 5a predicts that ISGylated nuclear NP molecules in 67 virus-infected cells would be associated with long chains of unconjugated NP molecules if the terminated NP oligomers are not degraded. To determine whether this is the case, we used ISG15GG A549 cells and purified ISGylated proteins by double-affinity selection of both cytoplasmic and nuclear fractions of cells that were pretreated with USP18 siRNA and IFN-β followed by infection with wt or 67 virus. Purification was performed under non-denaturing conditions to preserve any interaction between ISGylated NP and unconjugated NP. An immunoblot probed with NP antibody (Fig. 5e) showed that the small amount of ISGylated NP in the nucleus of 67 virus-infected cells (predominately NP-(ISG15)_2_) was associated with a large excess of unconjugated NP, as predicted by the termination model of Fig. 5a. Oligomerization of NP was retained after RNase digestion to remove the associated viral RNA, as shown previously by others^35^. ISGylated NP was not detected in the nucleus of wt virus-infected cells, and instead was bound to wt NS1B in the cytoplasm. In contrast, ISGylated NP in the cytoplasm of 67 virus-infected cells was not bound to 67 mutant NS1B. The presence of cytoplasmic ISGylated NP in 67 virus-infected cells indicates that ISGylation also inhibits nuclear import, consistent with the presence of two ISGylation sites (K69 and K77) in the N-terminal region of NP that contains the nuclear localization signal (Fig. 3b).

To provide definitive evidence that ISGylated NP inhibits viral RNA synthesis, we generated recombinant viruses expressing NP proteins in which one or more of the NP ISGylation sites is eliminated by K-to-R mutations, and determined whether the elimination of these ISGylation sites relieves the inhibition of vRNA synthesis in 67 virus-infected cells. Consequently, we identified a set of NP K-to-R mutations (at positions 156, 245, 279 and 478) that substantially relieved the IFN-β-mediated inhibition of vRNA synthesis in 67 virus-infected cells. Elimination of these four NP ISGylation sites increased HA vRNA production in 67 virus-infected cells from 50 to 81% of the level in wt virus-infected cells (Fig. 5f). SiRNA knockdown of ISG15 increased the HA vRNA production in 67 virus-infected cells to the level in cells infected with the 67 virus containing these four K-to-R mutations (Supplementary Fig. 5), demonstrating that the difference in vRNA levels is due to ISGylation of these four K residues. These results confirm that ISGylated NP is largely responsible for the inhibition of viral RNA synthesis in 67 virus-infected cells.

## Discussion

Although transfection assays indicated that the NS1B protein of influenza B virus inhibits ISGylation^23^, the present study demonstrates that ISGylation is not inhibited by the NS1B protein in influenza B virus-infected cells. Instead, the NS1B protein binds and sequesters ISGylated viral proteins. Using a recombinant virus that expresses a mutant NS1B protein defective in ISG15 binding, we elucidated a major consequence of the failure of the mutant NS1B protein to bind ISGylated viral proteins in infected cells. We showed that the viral NP protein is a primary target for ISGylation. We further demonstrated that the ISGylated NP proteins that are not bound to the mutant NS1B protein, although only a small percentage of the total NP proteins in mutant virus-infected cells, act in a dominant-negative manner to terminate the oligomerization of unconjugated NP molecules, resulting in incomplete NP oligomers containing ISGylated NP. This is the first demonstration that ISGylated viral proteins can act as dominant-negative inhibitors of the oligomerization of their much more abundant unconjugated counterparts in virus-infected cells.

Because NP oligomerization along viral RNA chains is required for the formation of viral RNPs that catalyse viral RNA synthesis^27^, the inhibition of NP oligomerization by ISGylated NP inhibits the formation of viral RNPs and hence inhibits viral RNA synthesis. This conclusion was further supported by our finding that elimination of a subset of the ISGylation sites of NP significantly relieves the inhibition of viral RNA synthesis. Inhibited viral RNA production results in inhibition of the synthesis of viral proteins, the primary targets for ISGylation, explaining the reduced level of ISGylation in 67 virus-infected cells. However, our results do not rule out the possibility that the other two viral ISGylation targets, M1 and NS1B, also have a role in inhibiting virus replication. Nevertheless, by identifying the sequestration of ISGylated viral proteins as a novel viral countermeasure against IFN-α/β-induced ISGylation, we establish that ISGylation can exhibit potent antiviral activity in human cells.

For NP proteins of both influenza A and B viruses, their oligomerization on the RNA template requires that the tail loop of each NP molecule is inserted into a cavity in the adjacent NP molecule^24^,^30^,^31^. Previous studies of the NP protein of influenza A virus (NP-A) showed that a NP-A mutant that is defective in either the loop or insertion site could function as a dominant negative mutant to inhibit the oligomerization of wt NP-A, thereby inhibiting the viral RNA synthesis catalysed by viral RNPs^32^,^33^. We would expect the same dominant-negative action by similar mutants of influenza B virus NP (NP-B) molecules, in light of the high structural similarity between the NP-A and NP-B proteins^24^,^31^. In fact, our results indicate that ISGylation of the NP-B protein probably impedes the function of its loop: ISGylation of K478, which is located at the base of the loop, is one of the four ISGylations that function in the dominant-negative inhibition of viral RNA synthesis. These results are consistent with the model shown in Fig. 5a and Supplementary Fig. 6 for the dominant-negative inhibition of NP-B polymerization by ISGylated NP-B. However, our results indicate that ISGylation sites in different regions of NP-B other than its loop and insertion site also apparently function in the inhibition of viral RNA synthesis, namely, K245 and K279, which are located in the RNA-binding surface, and K156, which is located in the body domain. It has been shown that mutant NP-A proteins that are defective in RNA binding can still be recruited to the viral RNA template by interacting with wt NP^32^. Consequently, any ISGylated NP-B molecules that are defective only in RNA binding might be recruited to nascent NP oligomers by wt NP. One possible consequence of incorporation of such ISGylated NP-B molecules is destabilizing the interaction of NP oligomers with viral RNA templates. The functional impact of the ISGylation at K156 is not known. Of note, the elimination of these four ISGylation site largely, but not completely, rescues ISGylation-mediated inhibition of viral RNA synthesis, suggesting that ISGylation of other K residues of NP may also impact viral RNA synthesis.

The preferential targeting of the NP protein for ISGylation in influenza B virus-infected cells differs from ISGylation targeting in cells infected with a 1972 H3N2 influenza A virus. The primary viral target in cells infected with this influenza A virus is the NS1A protein^13^,^14^, and ISGylation of the NP-A protein was not detected^13^. This difference between influenza A and B viruses can be attributed at least in part to the fact that influenza A viruses do not encode a protein analogous to the influenza B virus NS1B protein that directly counters the antiviral action of ISGylation. Consequently, it is likely that influenza A virus, but not influenza B virus, would be under selective pressure to develop other mechanisms to circumvent the antiviral activity of ISGylation. It was noted by others that the total lysine (K) content of the proteins of several influenza B viruses was ∼35% higher than that of several influenza A viruses^11^. The percentage of Ks in the NP-B proteins of all influenza B viruses is two-fold greater than that in the NP-A proteins of all influenza A viruses isolated from humans (Supplementary Fig. 7a), and none of these NP-A proteins contain Ks at the four sites corresponding to the ISGylation sites in the NP-B that function in the dominant-negative inhibition of viral RNA synthesis (Supplementary Fig. 7b). This difference between influenza A and B viruses is consistent with the hypothesis that ISGylation-driven evolution of influenza A viruses has led to a replacement of a substantial number of the Ks in their encoded proteins with Rs, thereby eliminating potential ISG15 modifications. In fact, ISGylation-driven evolution has likely occurred recently in H3N2 viruses. We previously showed that the K at position 41 of the NS1A protein is the primary target for ISGylation in cells infected with a H3N2 virus isolated in 1972, and that the replacement of this K with an R relieves the IFN-β sensitivity of the virus^13^. Such a K-to-R change occurred in the NS1A proteins of H3N2 viruses isolated after 1986, demonstrating that H3N2 viruses isolated after 1986 have acquired a mutation in nature that essentially eliminates their susceptibility to inhibition by IFN-β-induced ISGylation^5^.

ISG15 can regulate the human immune response in multiple ways^35^,^36^. There are two forms of ISG15 inside human cells, free and conjugated to target proteins. Recent studies found that free ISG15 could be proviral by interacting with, and thereby stabilizing USP18 to downregulate IFN-α/β signalling^18^. Subsequently it was hypothesized that the main function of ISG15 system in human cells is to inhibit the inflammatory response via free ISG15, whereas ISGylation primarily functions in the regulation of the level of free ISG15 and does not exhibit any significant antiviral activity^17^,^18^,^20^. However, we previously showed that ISGylation inhibits H3N2 influenza A viruses, and in the present study we clearly establish that IFN-β-induced ISGylation is a potent inhibitor of influenza B virus replication when the protective effect of the viral NS1B protein is eliminated. Free ISG15 does not have a significant role in this inhibition, as shown by our demonstration that the free ISG15 that remained after siRNA knockdown of the ISG15 E1 enzyme (UbE1L) did not inhibit viral protein production. Based on the present study and on the apparent ISGylation-driven evolution of H3N2 influenza A viruses, we think that it is reasonable to propose that IFN-α/β-induced ISGylation is a relatively ancient antiviral activity that viruses have overcome either by the action of a specific viral protein like NS1B that blocks the antiviral effects of ISGylation and/or by ISGylation-driven evolution of K-to-R mutations and/or other amino acid changes to eliminate ISGylation sites.

## Methods

### Viruses and cells

All the B/Yamanashi/166/98 NS1/NP mutant viruses were generated by reverse genetics method, employing the eight-plasmid bidirectional transcriptional system as described by Hoffmann *et al.*^36^. Virus stocks were grown in 9-day-old fertilized chicken eggs, and virus titers were determined using plaque assays in MDCK cells. All eight segments of the recombinant viruses were sequenced. A549 (CCL-185) and MDCK (CCL-34) cells were obtained from the American Type Culture Collection (ATCC) and maintained in Dulbecco's modified Eagles' medium (Invitrogen) supplemented with 10% fetal bovine serum. To establish the IFN-β-inducible S-3F-ISG15GG A549 cell line, a plasmid was constructed by inserting into the pGL3 vector a KpnI-XbaI DNA fragment composed of a S-3F-ISG15-encoding sequence linked to an ISG15 promoter element at its 5′ end. This DNA fragment was a product of PCR-mediated ligation of two smaller fragments: one fragment contains a ISG15 promoter sequence (bases -740 to 0) that was amplified from HeLa genomic DNA; the other fragment encodes S-3F-ISG15 generated by PCR-mediated addition of 3xFlag-S sequence to an ISG15-encoding sequence. The constructed plasmid (9 μg) was co-transfected with 1 μg of a linear puromycin selection marker (Clontech) into A549 cells. After selection with medium containing 1 μg ml^−1^ puromycin, individual clones were selected, expanded, and the clone showing the strongest IFN-β-dependent induction of S-3F-ISG15 as well as its conjugates was used for virus infection experiments. The control IFN-β-inducible S-3F-ISG15AA A549 cell line was generated using the same procedure.

### SiRNAs and antibodies

All siRNAs were ON-TARGET plus siRNAs purchased from Thermo Scientific. For each gene, three different siRNAs were initially tested, and the one with the highest silencing efficiency as indicated by immunoblotting was chosen for subsequent experiments. The sense sequences for ISG15, UbE1L, USP18-specific siRNAs are as follow: 5′-GCACCGUGUUCAUGAAUCUUU-3′ (ISG15), 5′-GCAUGGAGUUUGCUUUCUG-3′ (UbE1L), 5′-CUGCAUAUCUUCUGGUUUA-3′ (USP18). The control siRNA used was ON-TARGET plus non-targeting siRNA #1. To exclude any off-target effects, we also employed additional siRNAs for silencing ISG15 and USP18. The sense sequences of these siRNAs are as follow: 5′-GCAACGAAUUCCAGGUGUC-3′ (ISG15), 5′-GGAAGAAGACAGCAACAUG-3′ (USP18). Transfection of siRNA was carried out using Lipofectamine RNAiMax reagent (Invitrogen), using a final concentration of siRNA of 20 nM (10 nM for each siRNA if two siRNAs were transfected together). Commercial antibodies (Abs) used: anti-Flag M2 mouse Ab (Sigma #3165, dilution 1:3,000), mouse Ab against influenza B virus NP (Southern Biotech #10885-05, dilution 1:10,000 to 1:2,000), mouse Ab against influenza B virus M1 protein (Southern Biotech #10820-01, dilution 1:4,000 to 1:1,000), rabbit Ab against UbcH8 (Abgent #AP2118B, dilution1:1,000), and anti-β-actin Ab (Sigma #A1978, dilution 1:8,000 ), rabbit antibody against USP18 (Cell Signaling #4813, dilution 1:1,000), rabbit antibody against PARP (Cell Signaling #9542, dilution 1:1,000). The Ab specific for human ISG15 was kindly provided by Earnest Borden^37^. The expression of viral HA protein was detected using serum from ferrets subjected to influenza B virus infection, which was kindly provided by Jonathan McCullers^38^. To generate antibody specific for NS1B, the NS1B C-terminal domain of influenza B/Yamanashi/166/98 strain expressed and purified from bacteria (kindly provided by Gaetano T. Montelione) was sent to Harlan Bioproducts for Science (Indianapolis, IN) for rabbit polyclonal antibody production. The quantitation of western blots was performed using NIH ImageJ software and original western blot scans for (Figs 1, 2, 3, 4, 5) are provided in Supplementary Fig. 8.

### Assaying virus replication, viral RNA and protein synthesis

For multiple cycle growth, A549 cells were infected with 0.1 plaque-forming units (p.f.u.) per cell of wt or 67 mutant virus, followed by incubation at 34 °C in serum-free OPTI-MEM medium supplemented with 1.0 μg ml^−1^ N-acetylated trypsin. Aliquots of the medium were taken at interval of approximately 12 h and virus titers were determined using plaque assays with MDCK cells. For single-cycle virus replication, A549 cells were infected with 5–10 p.f.u. per cell of wt or 67 mutant virus, and were incubated at 34 °C in serum-free OPTI-MEM medium. Where indicated, cells were pretreated with 1,000 International Units (IU)  ml^−1^ of recombinant human IFN-β (Avonex, Biogen, Inc.) for 12–18 h before virus infection. For siRNA knockdown experiments, siRNAs were transfected into cells for 24 h before IFN-β treatment. At 24 h after virus infection, cells were collected and analysed using either immunoblots or real-time quantitative RT–PCR. For immunoblots, cells were lysed in high-salt RIPA buffer (50 mM Tris-HCl 7.5, 400 mM NaCl, 1 mM EDTA, 0.5% NP-40, 0.5% sodium deoxycholate, 0.1% SDS) supplemented with protease inhibitor and 0.5 units μl^−1^ benzonase (Sigma), then sonicated and cleared by centrifugation. The extracts were analysed by immunoblotting with the indicated Abs. For RT–PCR analysis, cells were harvested in Trizol reagent (Invitrogen), and total RNA was purified by Direct-zol RNA miniprep kit (Zymo research). Reverse transcription reactions were carried out using Transcriptor Reverse Transcriptase (Roche) with either oligo(dT) primer (for mRNA) or influenza B virus gene-specific primers (for vRNA). Real-time PCR was carried out on the ViiA 7 Real-time PCR system (Invitrogen). For the measurements of HA, M, NA, NP RNA, 2 × FastStart Universal Probe Master (Rox) (Roche) and Universal Probe Library (Roche) were used and human ACTB (Beta Actin) endogenous control (Applied Biosystems) was used as the internal control. Universal Probes (Roche) used were: #118 for HA and NP, #29 for M and #14 for NA. For the measurement of NS RNA, because of no suitable Universal Probe Library(Roche), SYBR Green based quantitative PCR with melting curve analysis was employed using Power SYBR Master Mix (Applied Biosystems). The same method was also applied to assaying NP and Actin RNA in the same experiments. The data were analysed using the comparative delta-delta Ct method. The primer sequences are provided in Supplementary Table 2.

### Co-immunoprecipitation

For analysis of NS1B-ISG15 interaction, 3F-S-ISG15GG A549 cells were first treated with the indicated siRNA for 24 h, followed by 16 h of treatment with 1,000 IU ml^−1^ of human IFN-β and then infection with 5 p.f.u. per cell of either wt or 67 mutant virus. The cells were harvested at 18 h after infection, and were extracted with cold RIPA lysis buffer (50 mM Tris-HCl pH 7.5; 200 mM NaCl; 2 mM MgCl_2;_ 0.5% NP-40; 0.5% sodium deoxycholate; 0.1% SDS) supplemented with protease inhibitor and 0.5 units μl^−1^ benzonase (Sigma). After brief sonication, the lysate was cleared by centrifugation at 16000xg for 10 min at 4 °C. The protein concentration of the extract was measured using the DC protein assay (Biorad), and an aliquot of the extract containing 700 μg of protein was used for co-immunoprecipitations. The extract was precleared by incubation with 50 μl Dynabeads Protein G (Invitrogen) for 2 h at 4 °C, and then incubated for 3 h with 50 ul Dynabeads Protein G (that had been preabsorbed with 10 μg of NS1B Ab). The beads were washed five times with RIPA buffer, and the bound proteins were eluted with SDS sample buffer, and analysed by immunoblots probed with NS1B or Flag Ab.

### Identification of ISGylated proteins in virus-infected cells

S-3F-ISG15GG A549 cells were treated with 1,000 IU ml^−1^of human IFN-β for 16 h, followed by infection with 5 p.f.u. per cell of wt virus. At 24 h after infection, cells were lysed in 5% SDS denaturing lysis buffer (150 mM Tris-HCl, pH 6.8; 5% SDS; 30% glycerol) at room temperature. The lysates were sonicated, mixed with nine volumes of cold (4 °C) dilution buffer (35mMTris-HCl, pH 7.4; 1% Triton X-100; 150 mM NaCl; 1% Triton X-100), and were then incubated with anti-Flag M2-agarose (Sigma) beads for 2 h at 4 °C. Flag-tagged proteins were eluted from the beads with 1 μg μl^−1^ 3 × Flag peptide, and the eluate was incubated with S-protein agarose beads (EMD Millipore) for 18 h at 4 °C. After extensive washing of the beads with the dilution buffer, proteins were eluted using the SDS sample buffer and subjected to SDS–polyacrylamide gel electrophoresis. The gel was analysed by colloidal blue staining. The four major protein bands detected using colloidal blue staining were cut out and sent to Taplin Biological Mass Spectrometry Facility, Harvard Medical School for mass spectrometry analysis. The protein bands were subject to in-gel trypsin digestion after being treated with iodoacetamide to alkylate free cysteine. To prevent alkylation of lysine residue, the alkylation reactions were carried out at room temperature and in the dark. Protein peptides were then extracted from the gel and analysed on a Q-Eactive mass spectrometer for high resolution MS and MS/MS data. The generated data were searched against human protein database and influenza B protein assembly with SEQUEST and the target-decoy strategy to filter data to a 1% false discovery rates. The quality of the identified di-Gly sites were assigned based on passing the 1% false discovery rate threshold and further verified by manual inspection of each spectrum.

### Assaying vRNP formation in the nucleus of infected cells

A549 cells in a 10 cm dish were transfected with the indicated siRNAs for 24 h, followed by treatment with 1,000 IU ml^−1^of human IFN-β for 16 h and then infection with 5 p.f.u. per cell of either wt or 67 mutant virus. At 16 h after infection, cells were collected by trypsinization and low-speed centrifugation. The cell pellet was extracted to yield cytoplasmic and nuclear fractions as described by Fodor *et al.*^39^ with minor modification. Briefly, the cell pellet was lysed for 10 min on ice with 2 ml of buffer A (0.2% Nonidet NP-40; 10 mM Tris-HCl, pH7.6; 10 mM KCl; 1 mM EDTA; 1 mM dithiothreitol (DTT)) supplemented with protease inhibitor. Nuclei were pelleted by centrifugation at 1,000*g* for 10 min, washed once with 1 ml of buffer A, and then resuspended in 360 μl of buffer B (0.5% Nonidet NP-40; 50 mM Tris-HCl, pH7.6; 200 mM NaCl; 0.2 mM EDTA; 1 mM DTT) supplemented with protease inhibitor and 200U ml^−1^ of RNaseOUT (Invitrogen). The nuclear extract was cleared twice by centrifugation at 1,000*g* for 10 min, and 200 μl of the clarified nuclear extract (∼50 μg) was loaded onto a discontinuous 33–70% glycerol gradient comprising 1 ml each of 33, 40, 50 and 70% glycerol prepared in glycerol gradient buffer (50 mM Tris-HCl, pH 7.6; 200 mM NaCl; 0.2 mM EDTA; 1 mM DTT) supplemented with protease inhibitor and 40 units per ml of RNaseOUT. Ultracentifugation at 245,419*g* was carried out in a Beckman SW55Ti rotor for 4 h. Fourteen fractions of 300 μl each were manually collected from the top to the bottom of the gradient, and analysed by NP immunoblots.

### Assay of association of ISGylated NP with NS1B and free NP

Cytoplasmic and nuclear fractions from USP18 siRNA-transfected, IFN-pretreated and virus-infected S-3F-ISG15GG A549 cells were prepared using the same procedure as described in assaying vRNP formation, except that RNaseOUT was replaced with Benzonase in presence of MgCl_2_ to remove RNA. The resulting fractions were subject to double-affinity selection, and analysed by immunoblots probed with NP or NS1B antibody.

### Data availability

The authors declare that the data supporting the findings of this study are available within the article and its supplementary information files, or available from the authors upon request.

## Additional information

**How to cite this article**: Zhao, C. *et al.* Influenza B virus non-structural protein 1 counteracts ISG15 antiviral activity by sequestering ISGylated viral proteins. *Nat. Commun.* 7:12754 doi: 10.1038/ncomms12754 (2016).

## Supplementary Material

Supplementary Information Supplementary Figures 1 - 8, Supplementary Tables 1-2 and Supplementary References
