# Supplementary material for: Influenza B virus non-structural protein 1 counteracts ISG15 antiviral activity by sequestering ISGylated viral proteins
Source: Nat Commun. 2016 Sep 2;7:12754. doi: 10.1038/ncomms12754 (PMC5025834; doi:10.1038/ncomms12754)
Supplement: Supplementary Information — Supplementary Figures 1 - 8, Supplementary Tables 1-2 and Supplementary References [file ncomms12754-s1.pdf]

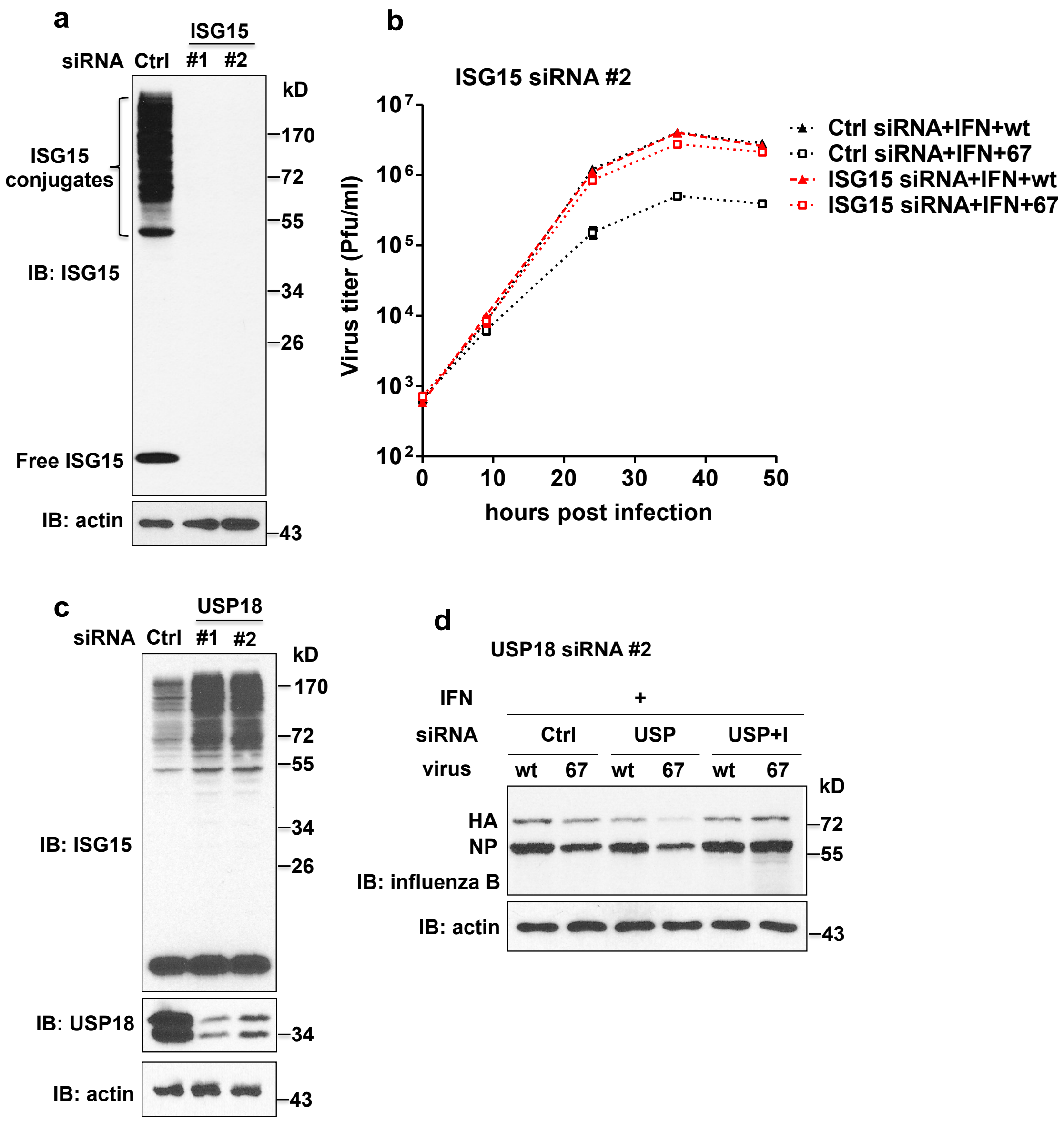

Supplementary Figure 1. Verification of siRNA knockdown experiments using additional siRNAs. (A) Knockdown efficiency of two different ISG15 siRNAs. A549 cells were transfected with 10nM of either control (ctrl) siRNA or the indicated ISG15 siRNA, followed by 24hour treatment with 1000IU/ml human IFN-β. Cell extracts were analyzed by immunoblots with the indicated Abs. ISG15 #1 siRNA is the siRNA used for the knockdown experiments described in the text. (B) ISG15 knockdown using ISG15 siRNA#2, like ISG15 siRNA#1, rescued the replication of 67 mutant virus (C) Knockdown efficiency of two different USP18 siRNAs as assayed by USP18 immunoblots of siRNA-transfected, IFN-treated A549 cells. USP18 siRNA #1 was used.in the experiments described in the text (D) USP18 siRNA #2, like USP18 siRNA#1, enhanced ISGylation and inhibited HA and NP protein production in 67 mutant-infected cells. Related to Figures 1 and 4.

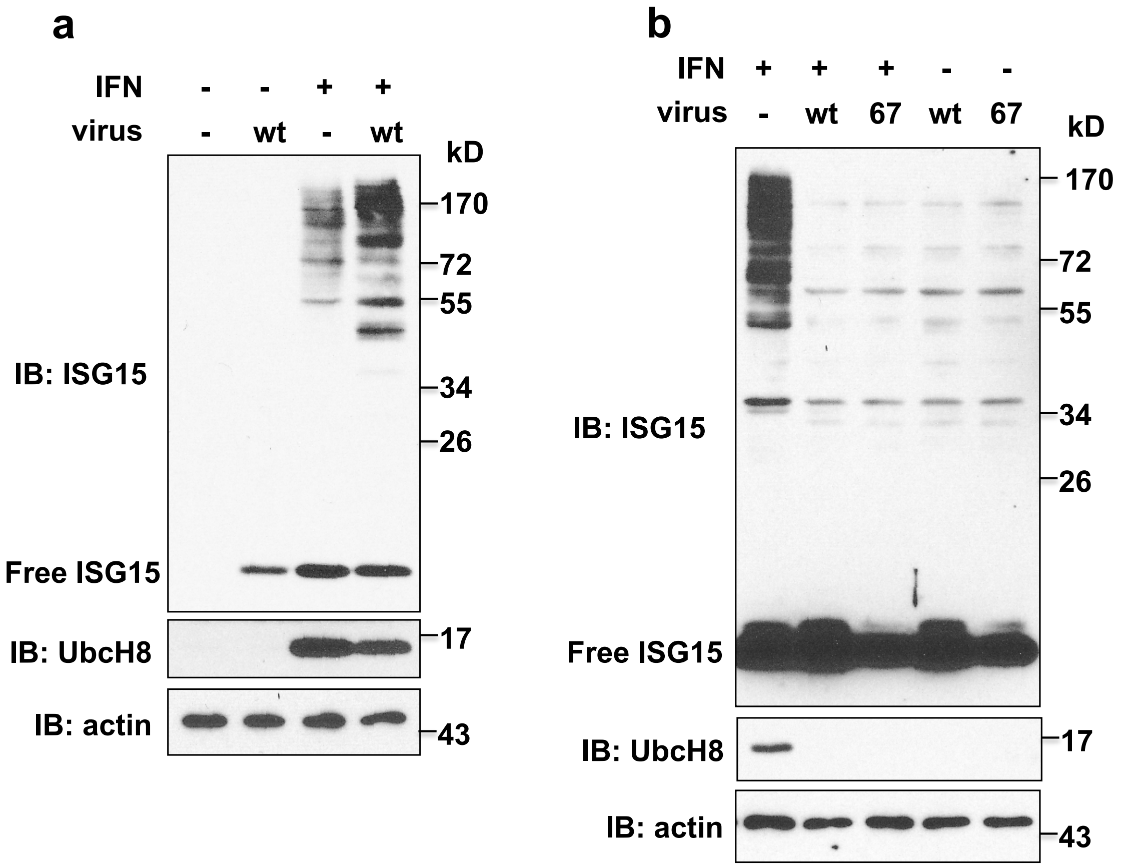

Supplementary Figure 2. ISGylation occurs only when IFN-β treatment precedes influenza B virus infection. (**A**) A549 cells were treated with1000IU/ml of human IFN-β for 12 hours or were not treated with IFN. Cells were then either mock infected, or infected with 5 pfu/cell of wt virus. Cell extracts isolated at 24 hours after infection or mock infection were analyzed by immunoblots probed with the indicated Abs. (**B**) Both wt and 67 mutant viruses inhibit IFN-induced synthesis of UbcH8. A549 cells were infected with 5 pfu/cell of wt or 67 mutant virus, or mock infected. At 6 hours after infection the cultural medium were replaced with fresh medium containing 1000IU/ml of human IFN-β. Cell extracts isolated at 24 hours after infection or mock infection were analyzed by immunoblots probed with the indicated Abs. Related to Figure 1.

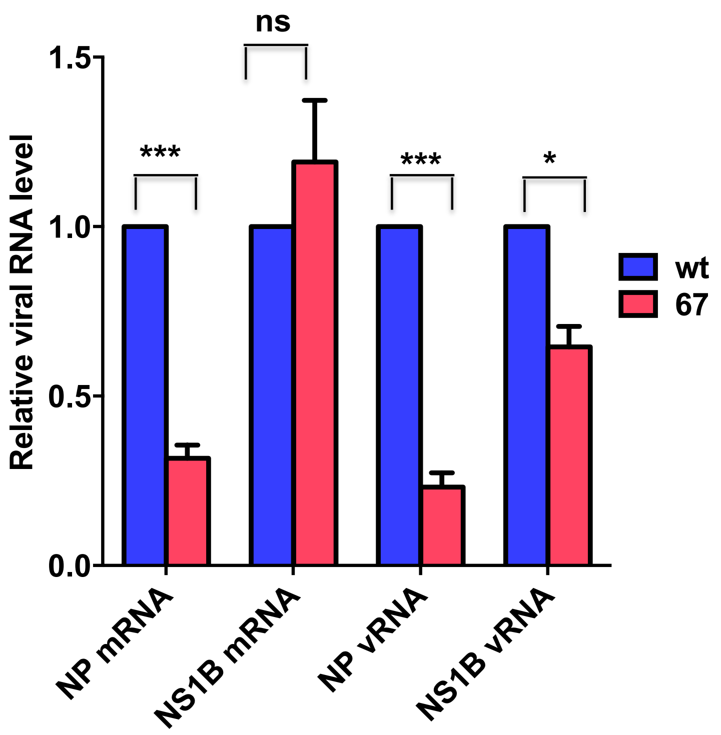

Supplementary Figure 3. Levels of mRNA and vRNA of NP and NS1B in wt and 67 infected cells. A549 cells were transfected with USP18 siRNA for 24hrs, followed by 16 hours of treatment with 1000IU/ml human IFN-β and subsequently infected with 5 pfu/cell of wt or 67 mutant virus. At 24 hours after infection, cells were collected, and the levels of NP mRNA, NS1B mRNA, NP vRNA, and NS vRNA were determined by RT–PCR, normalized to the level of β-actin mRNA. The ratios of the levels of these RNAs in 67-infected cells to the levels in wt-infected cells are shown. Results shown are mean +/- standard deviation of three independent experiments, with each measurement performed in triplicate. P values were calculated using 2-way ANOVA test. *** P< 0.001, * P<0.05, ns P>0.05.

A likely explanation for these results stems from the fact that NS1B mRNA, like the NS1A mRNA of influenza A virus, is synthesized predominantly at early times of infection. Experiments with influenza A virus has established that the early synthesis of NS1A mRNA is coupled with the early synthesis of NS vRNA, the template for the transcription of NS1A mRNA[1,2]. It is likely that this is also the case for NS1B mRNA and NS vRNA of influenza B virus. Consequently, a substantial amount of NS vRNA synthesis probably occurs prior to the time that ISGylation of NP results in the most effective inhibition of vRNA synthesis, resulting in little or no inhibition of NS1B mRNA.

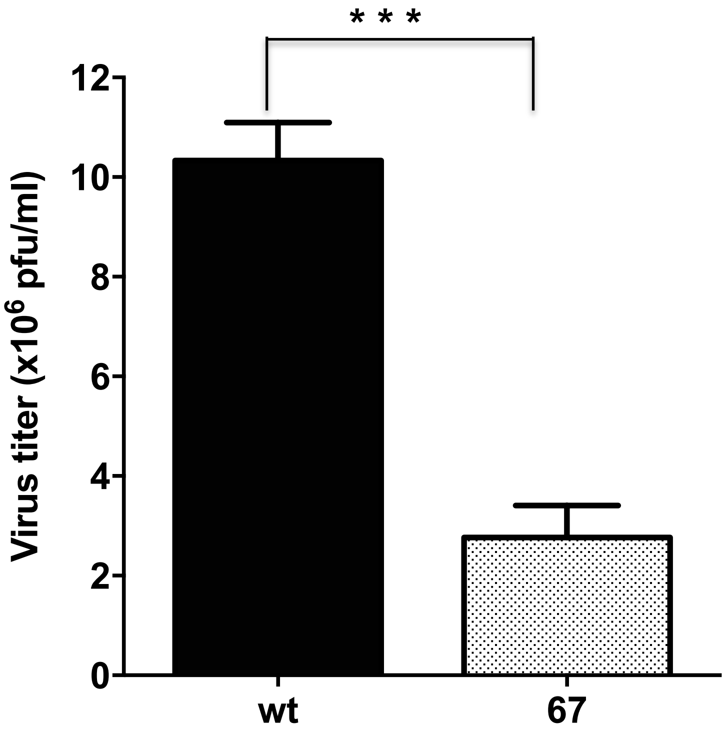

Supplementary Figure 4. Single cycle growth of wt and 67 mutant virus in A549 cells with IFN-β pretreatment. A549 cells were treated with 1000 IU/ml of human IFN-β for 16 hours prior to virus infection with 5 pfu/cell of either wt virus or 67 mutant virus. Bars show the standard deviation of triplicate assays of virus titers at 24 hours post infection. P value was obtained with two-tailed t-test. *** P<0.001.

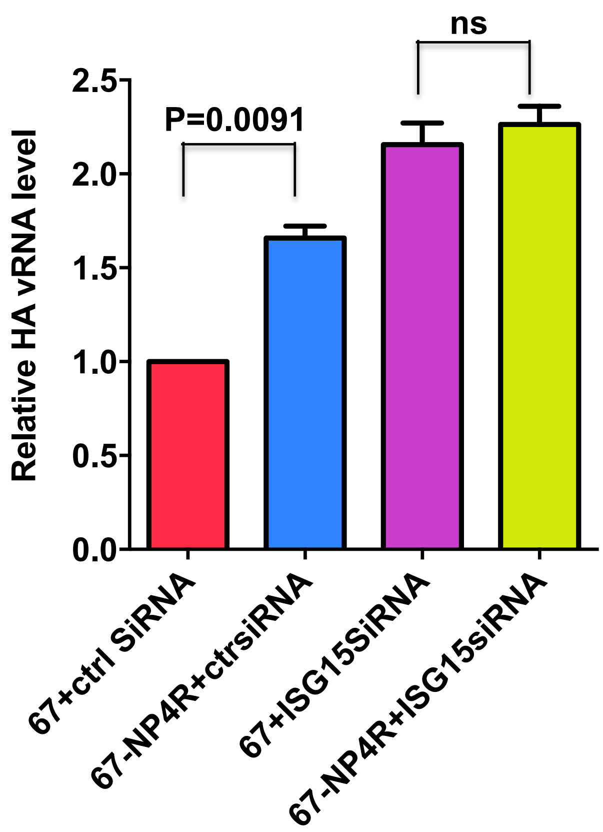

Supplementary Figure 5 .The effect of ISG15 knockdown on viral RNA synthesis of 67 mutant virus and 67-NP4R double-mutant virus in IFN-treated A549 cells. A549 cells were transfected with either control siRNA or ISG15 siRNA for 24 hours, treated with 1000IU/ml of human IFN-β for 16 hours prior to infection with the two mutant viruses at 5 pfu/cell. At 24 hours after infection, cells were collected, and the levels of the HA vRNA were determined by RT–PCR, normalized to the level of β-actin mRNA. Results shown are mean +/- standard deviation of three independent experiments, with each measurement performed in triplicate. P values were obtained with two-tailed t-test. ns, nonspecific.

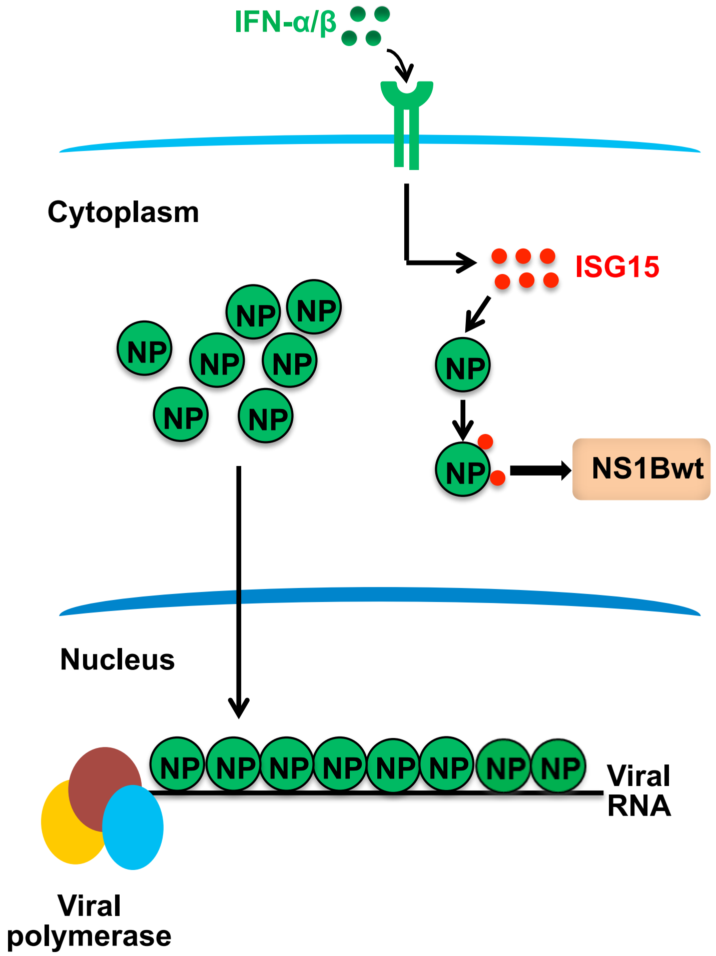

Supplementary Figure 6. Mechanistic model of the interplay between ISGylation and influenza B virus in human cells. In human cells infected with wt influenza B virus, the NS1B protein does not block the ISGylation process per se. The viral NP protein is thus ISGylated. However, the ISGylated NP proteins are not functional as they are bound and sequestered by the NS1B protein in the cytoplasm. When NS1B sequestration is eliminated, as demonstrated in the case of the 67 mutant virus, the ISGylatied NP proteins are free to enter the nucleus, where they terminate the oligomerization of NP molecules that lack ISG15 modifications, resulting in incomplete NP oligomers containing ISGylated NP. As a result, ISGylated NP inhibits the formation of viral RNPs that catalyze viral RNA synthesis.

a

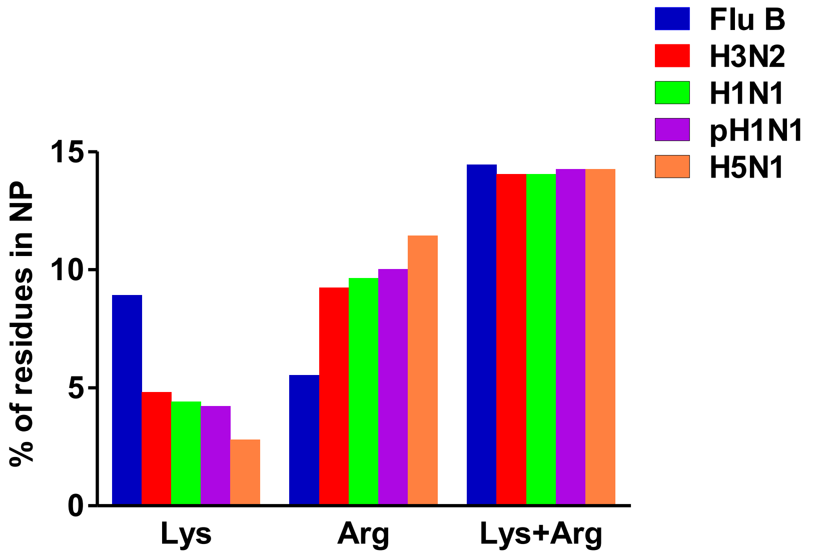

b

Position of ISGylated K in NP-B	156	245	279	478
Corresponding position in NP-A	98	184	221	422
H3N2	R	K	R	K
H1N1	K	K	R	K
pH1N1	R	K	R	R
H5N1	R	K	R	R

Supplementary Figure 7. Influenza B virus NP proteins contain more lysine residues than influenza A virus NP proteins. The sequences of NP protein from different influenza virus strain were obtained from the Influenza Research Database (IRD) and the consensus amino acid at individual positions was determined by Sequence Variation (SNP) analysis tool in IRD. Sequence alignment was constructed by using the PROMALS3D server[3]. (**A**) Percentage of Ks, Rs, and Ks plus Rs in NP proteins from influenza B viruses and influenza A virus strains that have infected humans. (**B**) Identity of the amino acids in influenza A virus NP proteins (designated as NP-A) at positions corresponding to the Ks that are identified as functional ISGylation sites in the influenza B virus NP (designated as NP-B).

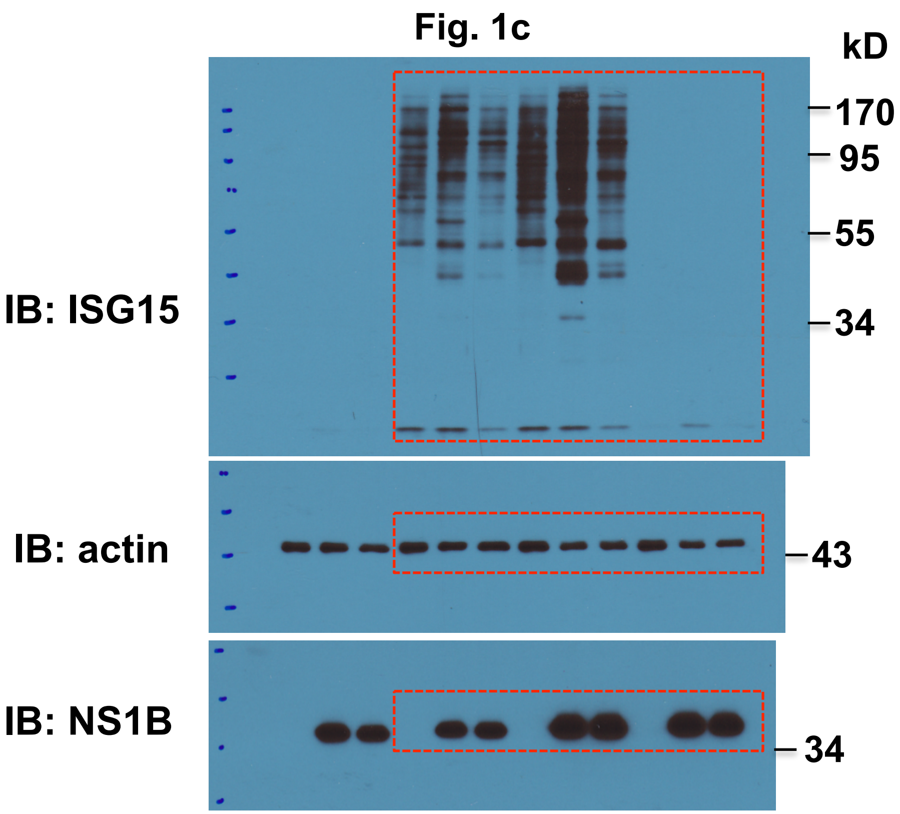

Supplementary Figure 8. Original western blot scans for Figures 1-5,

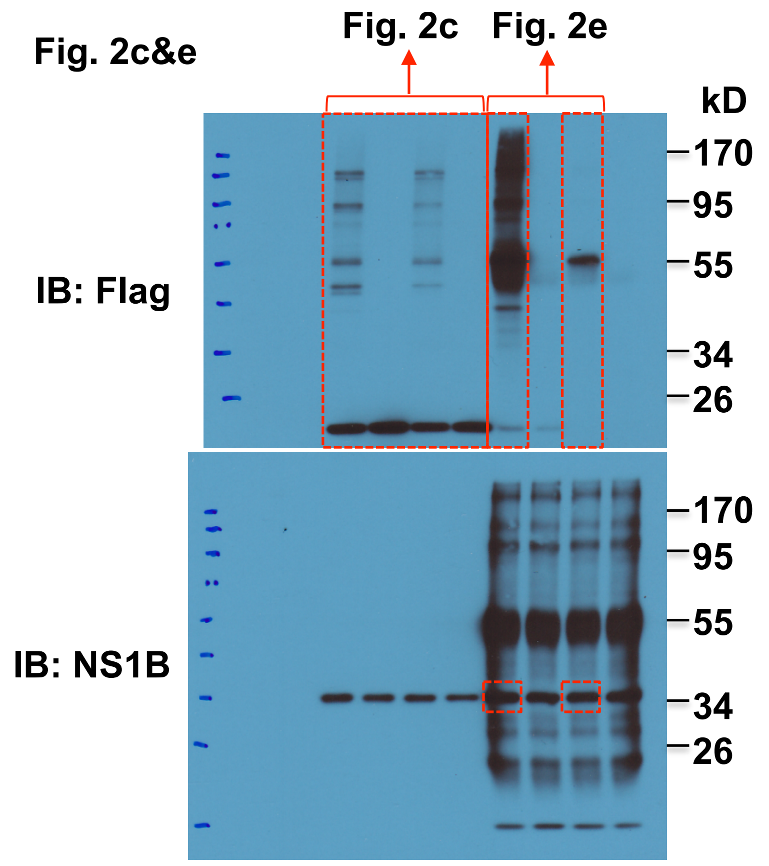

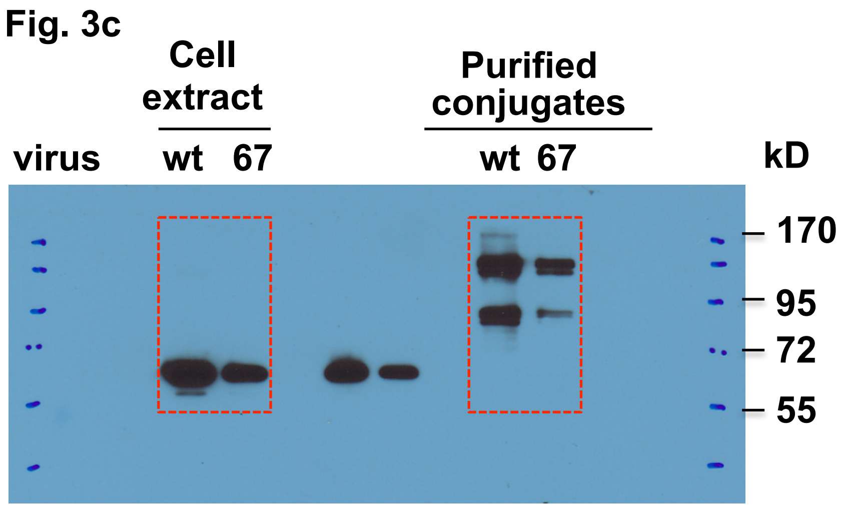

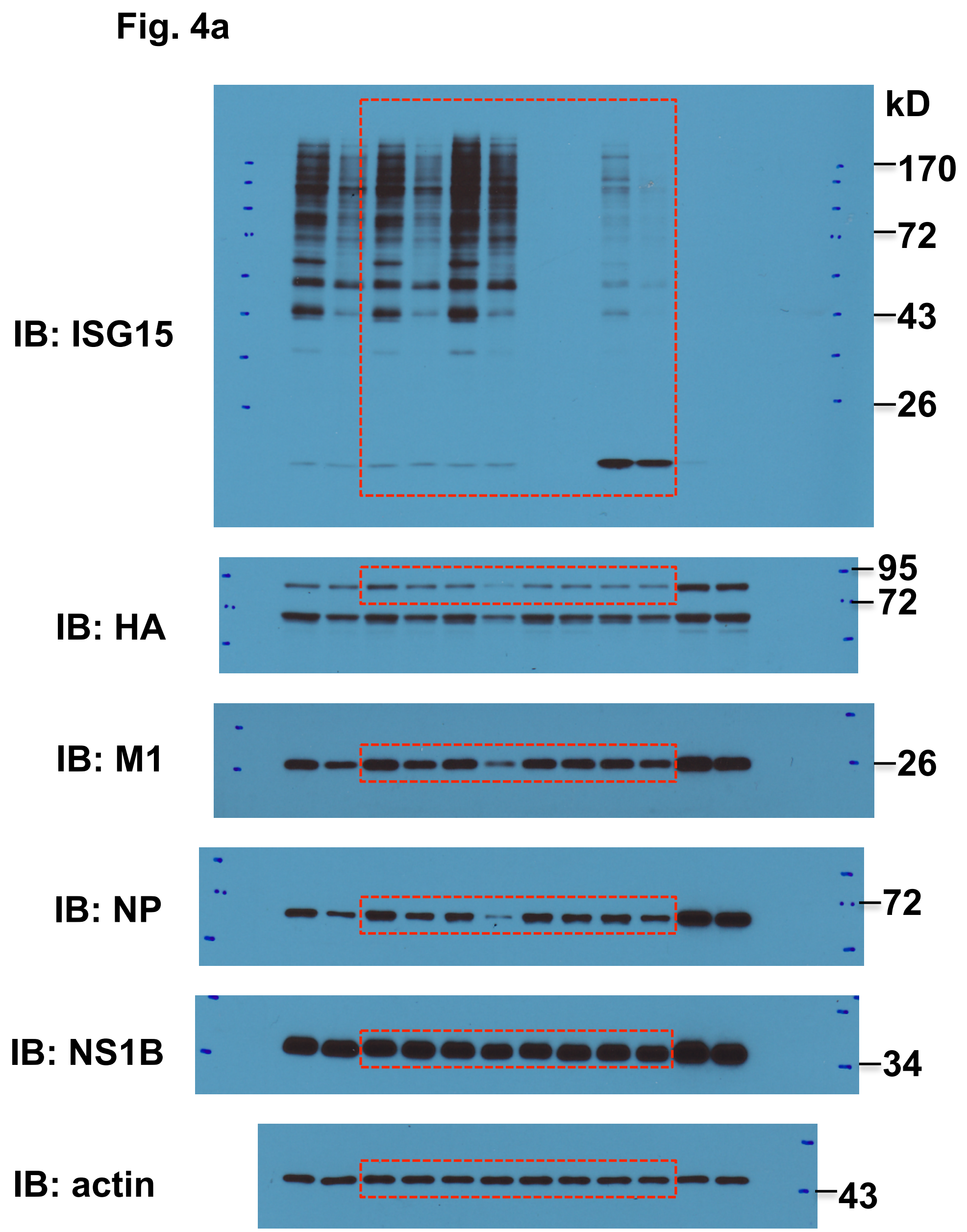

Supplementary Figure 8 (continued).

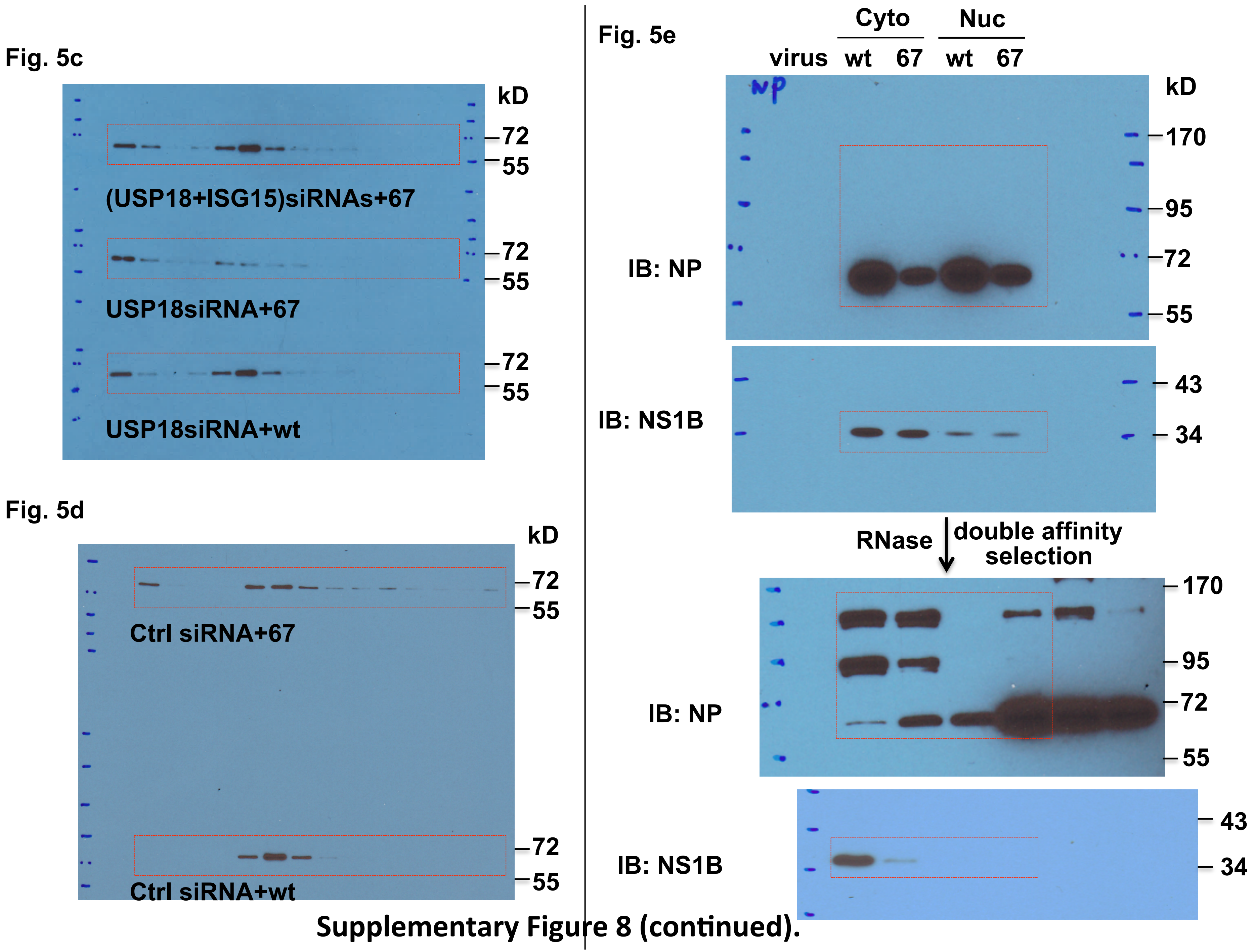

Supplementary Figure 8 (continued).

Supplementary Table 1. Identified ISG15-modified peptides by mass spectrometry analysis

dCn	Xcorr	Protein (residue)	Peptide Sequence
2.769	0.220	M1 (93-101)	K.KK#GLTLAER.K
2.703	0.601	M1 (94-101)	K.K#GLTLAER.K
5.042	0.788	NS1 (107-127)	K.SSSNSNCPK#YNWTDYPSTPGR.C
4.609	0.823	NS1 (107-127)	K.SSSNSNCPK#YNWTDYPSTPGR.C
3.172	0.215	NS1 (159-170)	K.IKEEVNTQK#EGK.F
2.398	0.580	NS1 (159-167)	K.IK#EEVNTQK.E
2.026	0.640	NP (69-76)	K.K#QTPTEIK.K
2.420	0.543	NP (69-77)	K.K#QTPTEIKK.S
3.296	0.572	NP (69-77)	K.K#QTPTEIKK.S
2.293	0.600	NP (77-85)	K.K#SVYNMVVK.L
3.121	0.266	NP (137-149)	R.DVK#EGKEEIDHNK.T
6.324	0.844	NP (448-482)	R.SGGNEVSGDGGSGQISCSPVFAVERPIALSK#QAVR.R
4.476	0.824	NP (448-482)	R.SGGNEVSGDGGSGQISCSPVFAVERPIALSK#QAVR.R
2.624	0.641	NP (150-159)	K.TGGTFYK#M*VR.D
3.008	0.692	NP (150-159)	K.TGGTFYK#M*VR.D
3.001	0.635	NP (150-159)	K.TGGTFYK#MVR.D
2.625	0.591	NP (150-159)	K.TGGTFYK#MVR.D
2.140	0.281	NP (171-179)	R.ITFLK#EEVK.T
3.937	0.676	NP (236-256)	R.RSGATGVAIK#GGGTLVAEAIR.F
4.936	0.716	NP (237-256)	R.SGATGVAIK#GGGTLVAEAIR.F
2.314	0.759	NP (237-256)	R.SGATGVAIK#GGGTLVAEAIR.F
5.434	0.703	NP (237-256)	R.SGATGVAIK#GGGTLVAEAIR.F
3.250	0.540	NP (275-285)	K.TAYEK#ILLNLK.N
3.194	0.492	NP (275-285)	K.TAYEK#ILLNLK.N
3.165	0.590	NP (275-285)	K.TAYEK#ILLNLK.N
2.888	0.532	NP (275-285)	K.TAYEK#ILLNLK.N
3.004	0.690	NP (288-304)	K.CSAPQQK#ALVDQVIGSR.N
3.609	0.709	NP (399-411)	R.VLSALTGTEFK#PR.S

Peptide identification was based on cross-correlation value (XCorr) and delta-cross correlation (dCn) values as indicated. M* denotes oxidized methione, and K# denotes lysine residue modified by diglycine, a remnant left by ISG15 after trypsin digestion. The identified peptides from band 1, as determined to be NP modified by two ISG15 moieties, was highlighted in red.

Supplementary Table 2. Primers for real-time PCR measurements

Target	Purpose	Sequence (5'-3')	Position(nt)
HA	Reverse Transcription	ATGAAGGCAATAATTGTACTACTCATG	1-27
	Real-time PCR	GTAACATCCAATGCAGATCGAA	42-63
		CACATGAGGTGAGTTTGACGA	81-101
M	Reverse Transcription	ATGTCGCTGTTTGGAGACACA	1-18
	Real-time PCR	GGAAAAGGAGAAGACGTCCA	580-599
		CAATACTCCAATGTTGCTTTGC	618-639
NA	Reverse Transcription	ATGCTACCTTCAACTATACAAACGTT	1-26
	Real-time PCR	GCAACAAAAGGGGTGACACT	229-248
		GGGCAAGATAAACGAGGGTA	274-293
NP	Reverse Transcription	ATGTCCAACATGGATATTGACG	1-22
	Real-time PCR	TCCAACATGGATATTGACGGTA	4-25
		TATTTCTTCCGGTGTTTTGTCA	42-63
	Real-time PCR*	TCGTAGCATGGTCGTTGTTAG	954-974
		GCTTCGTACCCAACCATAGAG	1056-1076
NS	Reverse Transcription	ATGGCGGACAACATGACCAC	1-20
	Real-time PCR	CTGCTGGAATTGAAGGGTTTG	281-301
		TCCTGGTGTTGAAGGGTAATC	358-378
ACTIN	Real-time PCR	GTCTTCCCCTCCATCGTG	88-105
		AGGGTGAGGATGCCTCTC	183-200

* Used in SYBR Green based quantitative PCR along with NS RNA measurement

Supplementary References

1. Smith, G.L. & Hay, A.J. Replication of the influenza virus genome. *Virology* **118**, 96-108 (1982).

2. Shapiro, G.I., Gurney, T., Jr. & Krug, R.M. Influenza virus gene expression: control mechanisms at early and late times of infection and nuclear-cytoplasmic transport of virus-specific RNAs. *J Virol* **61**, 764-73 (1987).

3. Pei, J., Tang, M. & Grishin, N.V. PROMALS3D web server for accurate multiple protein sequence and structure alignments. *Nucleic Acids Res* **36**, W30-4 (2008).
